# Effects of multimodal intensive rehabilitation program in lung transplant recipients: study protocol of a pragmatic randomized controlled trial

**DOI:** 10.3389/fmed.2025.1696184

**Published:** 2025-11-18

**Authors:** Siyuan Wang, Li Zhao, Beiyao Gao, Yajing Duan, Shuang Zheng, Peijian Wang, Wenhui Chen, Shan Jiang

**Affiliations:** 1 Department of Rehabilitation Medicine, China-Japan Friendship Hospital, Beijing, China; 2 National Center for Respiratory Medicine, Beijing, China; 3 State Key Laboratory of Respiratory Health and Multimorbidity, Beijing, China; 4 National Clinical Research Center for Respiratory Diseases, Beijing, China; 5 Institute of Respiratory Medicine, Chinese Academy of Medical Sciences, Beijing, China; 6 Department of Lung Transplantation, Center of Respiratory Medicine, China-Japan Friendship Hospital, Beijing, China

**Keywords:** lung transplant (LTx), rehabilitation, multimodal intensive, randomized controlled trial, study protocol

## Abstract

**Background:**

The main purpose of this study is to describe a protocol to compare the effectiveness of MIRP and conventional rehabilitation treatment.

**Methods/design:**

The study will be a single-blinded pragmatic randomized controlled trial involving patients (*N* = 100) with lung transplants. All patients will receive 6 weeks of treatment, while they will be divided into two groups and receive MIRP (*n* = 50) and conventional rehabilitation treatment (*n* = 50). Clinical functional assessment will be performed before, after intervention, and 3 months after the enrollment, respectively. Adults who have undergone unilateral or bilateral lung transplants and have just returned to the regular ward from the intensive care unit are eligible to participate.

**Discussion:**

The outcome of this study will provide a theoretical basis for clarifying intensive rehabilitation for lung transplants in future studies and clinical practice.

**Clinical trial registration:**

https://www.chictr.org.cn/showproj.html?proj=177086, identifier ChiCTR2200063538.

## Background

Lung transplantation (LT) is the ultimate treatment option for patients with end-stage lung disease (e.g., pulmonary fibrosis, bronchiectasis, and idiopathic pulmonary hypertension). Various complications and dysfunctions may occur after lung transplantation due to a variety of reasons, such as neuromuscular structural and functional abnormalities, the application of immunosuppressive drugs, and muscle wasting atrophy, which will bring about a series of problems, such as prolonged hospitalization, slow postoperative recovery, and increased therapeutic expenses (1). Previous studies have revealed that reduced exercise tolerance is common in postoperative lung transplant patients (2) and is associated with adverse health outcomes such as increased dyspnea and fatigue (3). Rehabilitation is a clinically proven effective physical factor to improve and reverse this quality-of-life decline (4, 5). Pulmonary rehabilitation (PR) is “a comprehensive intervention based on a thorough patient assessment followed by patient-tailored therapies that include, but are not limited to, exercise training, education, and behavior change, designed to improve the physical and psychological condition of patients and to promote the long-term adherence to health-enhancing behaviors” (6). The goal is to increase physiologic and functional status so that patients benefit from lung transplantation (7). New program models aimed at improving access and uptake have emerged in recent years, including tele-rehabilitation programs (8) and low-cost home programs (9), and the success of every rehabilitation program is evaluated in terms of whether the desired outcomes are achieved: these include improved exercise capacity, improved health-related quality of life, and reduced hospitalization rates (10).

Multimodal treatment is defined as applying several treatments simultaneously/concurrently or sequentially (one after another) to facilitate rehabilitation. The multiple modes used may include pharmacotherapy, devices, and behavioral/psychosocial interventions. The treatment was reported in treating different diseases: Parkinson’s disease (11), hemiplegic cerebral palsy (12), lung cancer surgery (13), spinal cord injury (14), etc. The structure and components of the multimodal exercise program were designed with reference to well-established protocols for patients with chronic obstructive pulmonary disease (COPD); one program included an 8-week (short-term) multimodal exercise program, containing inspiratory muscle training (IMT) plus neuromuscular electrical stimulation (NMES) (15); one program included 15 min of deep breathing exercises and 20–30 min of limb exercises (16); and one program combined health education, oral nutritional supplements, exercise, and oral testosterone for 90 days (17). The multimodal rehabilitation program (lasting 6 weeks) was developed in this study and encompasses postural support, respiratory exercises, exercise training with cycling and neuromuscular stimulation, daily living guidance for activities such as sitting balance and transfers, and early swallowing assessment and training to enhance oral feeding capabilities. Intensive rehabilitation aims to help patients regain function as soon as possible, improve quality of life, and increase survival and long-term quality of life through systematic rehabilitation interventions and treatments. Intensive rehabilitation was developed in this study, which was implemented 5–6 days per week and consisted of chest physical therapy techniques, inspiratory muscle strength training, and increasing exercise training, all under the supervision of a physiotherapist.

For this clinical study, we would assess 6 min walk distance (6MWD) as the primary clinical outcome. Previous studies have established an association between 6MWD and survival in patients after solid organ transplantation; shorter 6MWD is associated with increased risk of death, hospitalizations, and worse quality of life in patients evaluated for liver transplantation (18). There have been two key randomized clinical trials (RCTs) evaluating the impact of pulmonary rehabilitation (PR) on standard care after LT (19, 20). However, it remains difficult to conclusively attribute improvements in exercise capacity to the intervention itself. This is primarily due to the significant confounding factor of natural postoperative recovery. Furthermore, the minimal clinically important difference (MCID) for the 6MWT in an LT population has not been established, making it challenging to interpret the true clinical significance of any observed changes. This study aimed to contribute to this evidence base by evaluating a novel multimodal rehabilitation protocol.

Potential limitations of all previous RCTs that examined post-LT interventions include: (1) none used multimodal interventions, (2) the majority of studies were at high risk of bias, (3) lack of follow-up, and (4) some studies did not report critical outcomes such as 6 min walk distance. Thus, high-quality RCTs are needed. While previous studies with single interventions have demonstrated some effectiveness (10, 21), multimodal approaches have not been examined in individuals undergoing lung transplant (LTx). However, multimodal and multidisciplinary aftercare intervention was recommended after kidney transplantation (KTx); the intervention significantly improved outcomes after KTx (22). This study is a randomized controlled trial (RCT) designed to evaluate a novel MIRP against usual care in LTx recipients. Recognizing that conventional post-transplant rehabilitation often lacks the intensity and multidisciplinary focus needed to address multifaceted recovery challenges, MIRP integrates structured, high-frequency components—including chest physiotherapy, progressive strength training, and swallowing therapy—to target these evidence gaps directly. This report describes the research design and protocol for implementing and assessing this RCT.

### Study hypothesis and objective

This protocol was written in accordance with the Standard Protocol Items: Recommendations for Interventional Trials (SPIRIT) (23). Participants were recruited from the Lung Transplantation Department of the Center of Respiratory Diseases, China-Japan Friendship Hospital, China. The multimodal intensive rehabilitation program is designed to comprehensively improve patients’ respiratory muscle function, voice and swallowing function (24), and limb strength, thereby shortening hospital stay and improving the quality of life. The primary outcome was to analyze the effects of MIRP on the 6 min walking distance in lung transplant recipients. Secondary outcomes were (1) short-term change in health-related quality of life; (2) body skeletal muscle mass and skeletal bone condition; (3) respiratory muscle function and lung function; (4) psycho-emotional states; (5) the state of swallowing function and nutrition; (6) Clinical Frailty Scale; and (7) adverse events related to rehabilitation.

## Method/design

### Study design

The inclusion criteria are as follows: (1) recipients of unilateral or bilateral lung transplantation and (2) age between 18 and 75 years.

The exclusion criteria are as follows: (1) inability to obtain informed consent from the patient or a legal representative; (2) severe postoperative complications, such as anastomotic leakage, pulmonary embolism, or other major complications requiring prolonged mechanical ventilation or reoperation; (3) dependence on invasive mechanical ventilation or endotracheal intubation when transferred back to the general ward; (4) hemodynamic instability, defined as mean arterial pressure (MAP) < 60 mmHg or > 110 mmHg, heart rate < 50 bpm or > 130 bpm, or the requirement for high-dose vasoactive agents (e.g., norepinephrine > 0.1 μg/kg/min); (5) insufficient respiratory reserve, defined as a ratio of arterial oxygen partial pressure to inspired oxygen fraction (P/F ratio) ≤ 300, or oxygen saturation (SpO₂) ≤ 90% while receiving an inspired oxygen fraction (FiO₂) ≥ 0.6; (6) unstable cardiovascular conditions, including recent myocardial infarction, uncontrolled hypertension, unstable angina, severe arrhythmias, or advanced heart failure; (7) neurological or psychiatric disorders that interfere with rehabilitation participation, such as uncontrolled seizures, severe cognitive impairment, or severe psychiatric illness; and (8) musculoskeletal limitations that preclude active participation in rehabilitation, such as severe arthritis, recent fractures, or recent orthopedic surgery. The withdrawal criteria are as follows: (1) patient or legal representative requests to withdraw consent; (2) development of severe medical complications after enrollment that preclude safe participation in the rehabilitation program; (3) clinical deterioration requiring transfer to the ICU or re-initiation of invasive mechanical ventilation; (4) any adverse events deemed by the treating physician to require discontinuation of the intervention for patient safety; and (5) non-compliance with study procedures that prevents adequate data collection.

### Participant timeline

**Figure 1:**
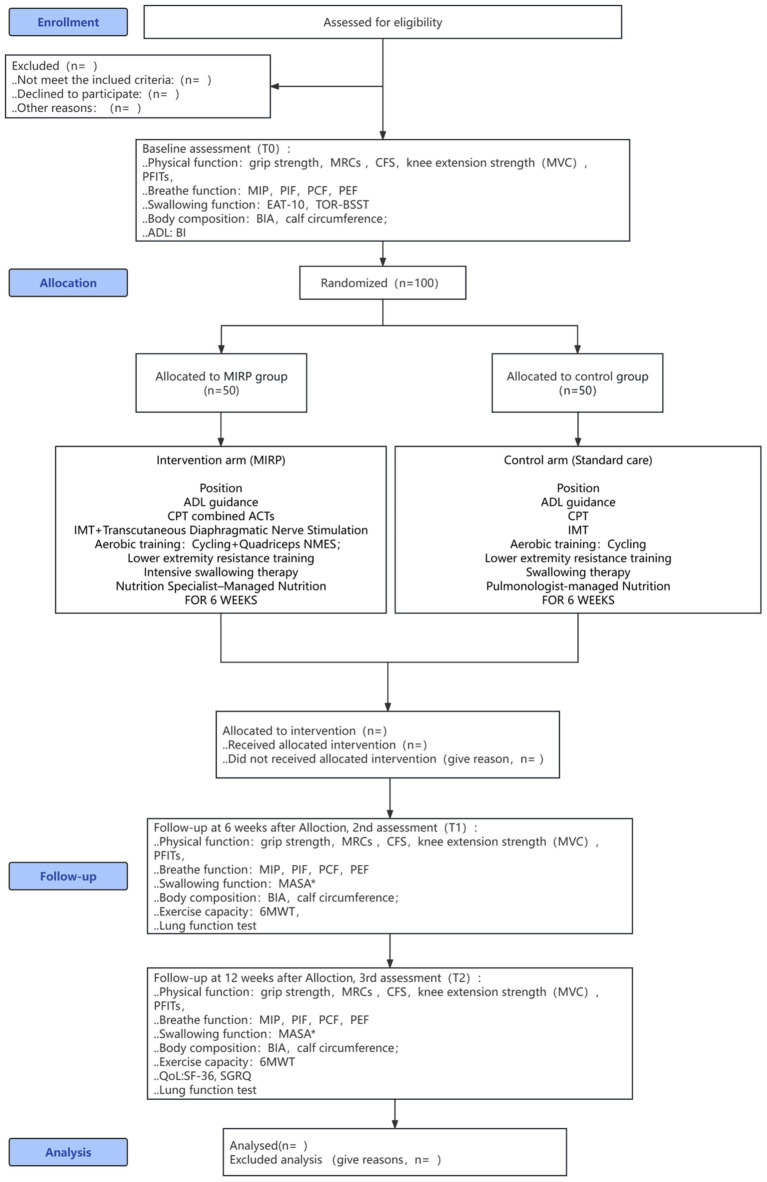
Study schematic diagram. MRCs, Medical Research Council sum score; MVC, maximum voluntary contraction (specifically for knee extension strength); PFITs, physical function in intensive care test—scored; MIP, maximum inspiratory pressure; PIF, peak inspiratory flow; PCF, peak cough flow; PEF, peak expiratory flow; EAT-10, 10-item Eating Assessment Tool; TOR-BSST, Toronto Bedside Swallowing Screening Test; MASA, Manchester Orofacial Assessment (likely a specific version); body composition assessment; BIA, bioelectrical impedance analysis; activities of daily living (ADL) assessment; BI, Barthel Index; MIRP: (Context suggests a specific rehabilitation protocol, e.g., multimodal intensive rehabilitation program. The exact term was not spelled out in the image). NMES, neuromuscular electrical stimulation; Quadriceps NMES, neuromuscular electrical stimulation for the quadriceps muscle; breath muscles NMES, neuromuscular electrical stimulation for the respiratory muscles; IMT, inspiratory muscle training; ACTs, active cycle of breathing techniques; OPEP, oscillating positive expiratory pressure; ACBT, active cycle of breathing technique; 6MWT, 6-min walk test (measures exercise capacity); QoL, quality of life; SF-36, 36-Item Short Form Health Survey; SGRQ, St. George’s Respiratory Questionnaire.

This study is ongoing and started in September 2024, ending in December 2025. The schedule for enrollment, interventions, and assessments is presented in Figure 1.

### Sample size

The significance level is set at 0.05, corresponding to a Z-value of 1.96. The power of the test is 90%, corresponding to a Z-value of 1.28. The assumed mean 6MWD values (339 m for the MIRP group and 320 m for the control group) and standard deviations for both variables are given as 20 m, and the resulting effect size used for the sample size calculation was derived from our center’s preliminary pre-study data. These estimates were based on an initial analysis of a small cohort of patients who underwent the intervention protocol prior to the initiation of this formal randomized controlled trial. Finally, there is a non-inferiority margin, denoted as *Δ* = 30, which requires 35 patients per group. A 5% dropout rate has also been accounted for, resulting in the target of 50 participants per group (100 in total).

### Recruitment

Patients who have just returned to the regular ward from the intensive care unit are screened for eligibility within the first 24 h, and informed consent is obtained from each participant by trained research staff. For patients who do not initially meet eligibility criteria during this first 24 h screening, the reasons for exclusion will be recorded. These patients will be re-assessed every 24 h. If they meet the eligibility criteria within 3 days, they will be enrolled in the study. If they meet the criteria after 3 days, they will not be enrolled or included in the study analysis; however, they will continue to receive standard rehabilitation care and undergo regular clinical assessments. Strategies to raise trial awareness have been implemented. Patients are informed about the postoperative rehabilitation program during preoperative discussions, provided with a brief rehabilitation information sheet, and exposed to educational posters permanently displayed in the ward. A small group of lung transplant recipients and their caregivers reviewed the patient information posters and the short information sheet. Their feedback was incorporated to ensure the materials were clear, understandable, and relevant to the target patient population.

### Common rehabilitation methods for both groups

**Figure 2:**
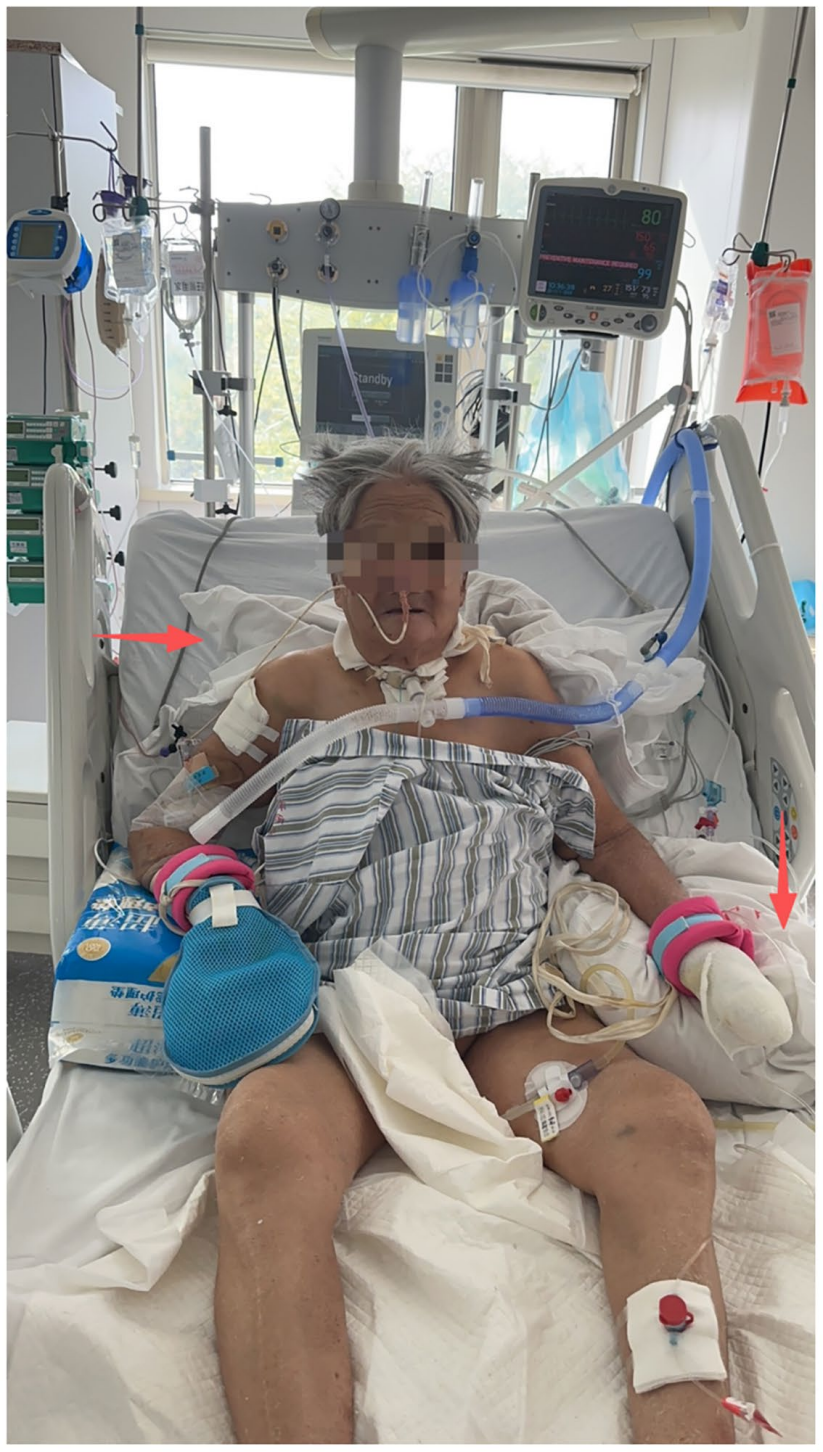
Positioning of a patient during sitting training. The upper left arrow points to a supportive pillow behind the patient’s back, facilitating an upright trunk position. The upper right arrow shows the support under the elbow against the bed rail, which helps stabilize the torso. The overall arrangement allows for the simultaneous use of nasal oxygen/enteral tubes, intravenous access, and monitoring leads without interference.

1. Positioning was used to promote respiratory mechanics while ensuring patient safety. Caregivers performed all positioning interventions in bed, including guided adjustments of the patient’s hips, scapula, and trunk to optimize lung expansion. Sitting out of bed or transferring to a bedside chair was performed under the supervision of a physiotherapist until the therapist confirmed it was safe and authorized the caregiver to perform it independently. Patients maintained an upright position in bed for 15–30 min, at least four times daily, with gradual extension up to 2 h as tolerated. Blood pressure and symptoms were monitored to ensure safety during the intervention. To encourage early mobilization, patients were educated to reduce prolonged bed rest; for instance, if a patient had been resting in bed for 2 h, they were encouraged to attempt sitting upright. Position changes were also performed to reduce skin pressure until patients could maintain the upright position independently (Figure 2).
2. Activities of daily life guidance involve patients turning over and getting up, sitting balance, sitting center of gravity transfer, bed-to-wheelchair transfer, from sitting to standing, walking, and other movements for guidance and training.

The supervised intervention concludes at 6 weeks for all participants, at which point they are provided with a standardized, personalized plan for unsupervised rehabilitation. This plan is continued until the 3-month assessment, regardless of whether the patient has been discharged or remains in the hospital.

### Intervention arm (MIRP)

1. Chest Physical Therapy Combined with Airway Clearance Techniques. In the intervention arm, chest physical therapy (CPT) is combined with airway clearance techniques (ACTs) as an integrated intervention. During each session, trained physiotherapists place their hands in a semicircle on the lower edge of the patient’s ribs to provide manual assistance. Guided by these hands-on techniques, patients perform deep inhalation with a 2–4 s breath hold as part of thoracic expansion techniques (TETs). During exhalation, a low-pressure oscillatory positive expiratory pressure (OPEP) device (10 cmH₂O, 12–14 Hz) is used, while the physiotherapist provides rib cage compression (RECC) and manual chest wall vibration to facilitate mucus clearance. Each set is repeated 3–5 times, with a 1 min rest between sets, and 5–8 sets are performed per session, five times per week.
2. Inspiratory Muscle Training Transcutaneous Diaphragmatic Nerve Stimulation. IMT is performed using a threshold device, with an initial load set at 50% of the patient’s maximal inspiratory pressure (MIP). Each session consists of 30 repetitions conducted five times per week. The training intensity is progressively adjusted according to patient tolerance and improvement in respiratory strength. Transcutaneous diaphragmatic nerve stimulation is applied using surface electrodes. The active electrode is positioned at the lateral edge of the sternocleidomastoid muscle, at the lower third, while the reference electrode is placed at the second intercostal space along the mid-clavicular line. Stimulation parameters are set at 40 Hz, with current intensity adjusted to a tolerable range of 10–30 mA. Each session is conducted under the supervision of a physiotherapist to ensure safety and optimize diaphragmatic activation.
3. Aerobic training combined with Neuromuscular Electrical Stimulation. Recumbent cycling is for bedridden patients, starting at 10 min and gradually increasing to 20 min. Patients are encouraged to actively participate, targeting an intensity of 4–5 on the Borg Fatigue Scale, three times a week. Patients who can leave the bed and transfer to a wheelchair should switch to seated cycling, with continuous exercise for 20 min per session, at an intensity of 4–5 on the Borg Fatigue Scale, five times a week. The NMES is incorporated into the recumbent cycling training, using modulated medium-frequency electrical stimulation, which is delivered through 4 × 4 cm self-adhesive gel electrodes. A bipolar electric current was applied with a frequency of 75 Hz and a pulse duration of 50 μs (5 s on and 5 s off).
4. Lower extremity resistance training: It uses freehand resistance methods, targeting the knee extensors, hip extensor muscle group, and hip abductor external rotator muscle group. Each exercise is performed for 1–3 sets, with 8–12 repetitions per set, twice a week. The principle of freehand resistance is to use the maximum resistance that allows the full range of joint movement, twice a week.
5. Intensive swallowing therapy: It consisted of pharyngeal electrical stimulation (PES) combined with traditional swallowing exercises, both administered by a certified speech-language pathologist (SLP). The swallowing exercise regimen, tailored based on the individual’s Kubota drinking test or volume-viscosity swallow test (VVST) results, included sensory and motor techniques such as thermal-tactile stimulation, effortful swallow, and the Mendelsohn maneuver. This combined therapy (PES + exercises) was conducted for 30 min per session, three times per week.
6. Nutrition Specialist–Managed Nutrition: Patients are managed directly by a nutrition specialist. The specialist closely monitors actual nutritional intake, including calories, protein, and fluid balance, and evaluates laboratory indicators (e.g., albumin, prealbumin, and electrolytes) to assess nutritional status. Based on these assessments, the nutrition plan is dynamically adjusted to meet individual patient needs, including modifying nutrient composition, supplementation, or feeding routes. The nutrition specialist also provides ongoing guidance to the clinical team to ensure adherence to the plan, thereby optimizing postoperative nutritional support.

### Control arm (standard care)

1. Chest physical therapy techniques are administered by physical therapists. The therapist places their hands in a semicircle on the lower edge of the patient’s ribs, first following the patient’s breathing frequency and amplitude. Upon the patient’s long exhalation command, the therapist gently applies downward and inward pressure on the patient’s thorax as they exhale. Then, the therapist releases the pressure and instructs the patient to inhale deeply, using their fingers to slightly lift the thorax upward and outward to promote deep inhalation. This procedure is repeated 3–5 times per set, with a 1 min break between sets, and 5–8 sets are performed per session, five times a week.
2. Inspiratory Muscle Training is performed using a threshold device, with an initial load set at 50% of the patient’s maximal inspiratory pressure (MIP). Each session consists of 30 repetitions, conducted five times per week. The training intensity is progressively adjusted according to patient tolerance and improvement in respiratory strength.
3. Aerobic training includes two types of bicycle training: horizontal and seated. For bedridden patients, horizontal cycling starts at 10 min and increases to 20 min as tolerated, with an intensity of 4–5 on the Borg Fatigue Scale, performed three times a week. Patients who are able to transfer from bed to wheelchair switch to seated cycling for 20 min per session at the same intensity, also three times a week.
4. Lower extremity resistance training involves using freehand resistance to target knee extensors, hip extensors, and hip abductor external rotators, with each exercise performed for 1–3 sets of 8–12 repetitions, once a week. The resistance should be the maximum that allows the full range of motion.
5. Swallowing therapy comprises traditional swallowing exercises administered by a certified speech-language pathologist (SLP). The exercise regimen was similarly personalized based on findings from the Kubota drinking test, or VVST, focusing on techniques such as thermal-tactile stimulation, effortful swallow, and Shaker exercises. This standard therapy was conducted for 30 min per session, twice per week.
6. Pulmonologist–managed Nutrition (PN): After receiving training from a lung transplant–specialized nutritionist, the pulmonologist develops a nutrition plan for each patient. The plan mainly includes the route of nutritional support, target nutritional intake, and basic monitoring indicators.

### Outcome measurement

The selection of assessment time points was based on clinical relevance and the study objectives. The 6-week assessment was chosen to evaluate the short-term efficacy and immediate impact of MIRP upon completion of the supervised, intensive intervention phase. The 3-month assessment serves as a key mid-term follow-up point to determine the sustainability of initial gains and to capture clinically significant outcomes as patients transition into the maintenance phase of recovery. All outcome assessments will be conducted by trained research assistants who are blinded to the participants’ group allocation. The primary outcome measure of treatment failure is assessed at 3 months after recruitment. The following are assessed at 6 weeks and 3 months after recruitment, including:

1. Short-term change in health-related quality of life, based on the modified Barthel Index and 36-Item Short Form Health Survey (SF-36).
2. Body skeletal muscle mass and bone mineral density were assessed using dual-energy X-ray absorptiometry; limb muscle strength was evaluated by measuring isometric knee extension strength using a handheld dynamometer (expressed in kg); limb dimensions were determined by measuring the circumference of the thigh and calf using a non-elastic tape measure; global muscle function was scored using the Medical Research Council (MRC) Scale for muscle strength, which provides a composite score (ranging from 0 to 60) based on the manual testing of six key muscles bilaterally.
3. Respiratory muscle and lung function were comprehensively evaluated using the following objective measures: maximum inspiratory pressure (MIP) to assess inspiratory muscle strength, standard spirometry for lung function parameters, and diaphragmatic ultrasound to quantify diaphragmatic excursion and thickening fraction (TFdi) as key indicators of diaphragmatic performance. Patient-reported dyspnea was assessed using the St. George’s Respiratory Questionnaire (or Dyspnea Rating Scale).
4. Psycho-emotional states, based on the Anxiety Self-Rating Scale and Depression Self-Rating Scale.
5. The state of swallowing function and nutrition, based on the Toronto Dysphagia Bedside Screening Test, 10-item Eating Assessment Tool (EAT-10) Swallowing Screening Scale, grade, roughness, breathiness, asthenia, and strain (GRBAS) grading scale for hoarseness of voice, and the Mann Assessment of Swallowing Ability Scale (MASA).
6. Adverse Events Monitoring: All adverse events (AEs) occurring during or within 24 h of a rehabilitation session were recorded. Examples of monitored AEs included musculoskeletal pain beyond typical soreness, falls, excessive dyspnea, cardiac symptoms, oxygen desaturation >4%, and skin irritation from equipment.

### Randomization and allocation concealment

Eligible patients are randomized using an automated system, with randomly permuted blocks, 1:1 to the intervention and standard care groups. This use of an automated randomization system and restricting access to the randomization sequence to only the trial statistician ensures that allocation concealment is maintained.

### Blinding

Due to the pragmatic nature of this trial, participants, care providers, and the research team are not blinded to the allocated treatment. Furthermore, patient representatives advised that participants were highly likely to reveal their treatment allocation to outcome assessors, and therefore, any attempt to blind this group would also be subverted.

### Data collection methods and management

Trial data, including study-specific worksheets and the patient questionnaires, are entered onto a case report form on paper. Data are processed as per the study-specific data management plan. Patient confidentiality is maintained with patients only identified by their assigned unique trial identifier and initials, and all databases are password-protected. Compliance with the protocol and the implemented amendments is carefully reviewed. All protocol amendments to date have received the appropriate regulatory and ethical approvals prior to electronic dissemination to investigators, with appropriate training provided by the China-Japan Hospital Science and Technology Center.

### Dissemination

The study findings will be published in international peer-reviewed journals and presented at both national and international meetings and to appropriate patient groups, with authorship granted to those who have made a substantive intellectual contribution to the study. Requests for data sharing will be reviewed on an individual basis by the chief investigators and the trial management group.

## Discussion

Researchers have described MIRP, an intensive pulmonary rehabilitation (PR) program tailored for pediatric patients after lung transplantation. A case report (25) suggested its potential feasibility and indicated possible improvements in functional exercise capacity, lung function, and quality of life in a 10-year-old recipient. However, as this was a single-case study with inherent limitations such as selection bias and lack of generalizability, further evidence from larger cohort studies or controlled trials is needed to conclusively establish the efficacy and safety of implementing such intensive PR programs in the broader pediatric lung transplant population. Moreover, the mechanisms underlying improvement in exercise capacity with MIRP among patients after LT are not well understood. With regard to the influential factors, peripheral muscle abnormalities have been shown to be the predominant limiting factor to exercise capacity in LT recipients; in addition, the original lung disease and pathophysiology may also influence an individual’s exercise capacity and physical activity (26). Recent studies have shown that exercise contributes to significant improvements in central pulmonary perfusion and peripheral skeletal muscle function that are beneficial for improvement in exercise capacity. This is probably because improved pulmonary perfusion is likely to lead to improved oxygenation and cardiac output and, thus, improvement in exercise tolerance and cardiorespiratory fitness (9). Moreover, Memme et al. (27) reported that exercise could help adjust the volume, structure, and capacity of mitochondria; thus, MIPR may help alleviate the muscle atrophy and damage to the respiratory function of mitochondria resulting from long-term immobilization before and after LT and the use of immunosuppressive drugs.

This study will provide new foundational data to determine whether an integrated model combining chest physical therapy, rehabilitation exercise training, nutrition, and swallowing management can improve physical endurance and quality of life after lung transplantation (24, 28). As intended by Chinese medical organizations, this study has the potential to optimize lung transplantation care in China, and all elements that are positively evaluated in our study have the potential to become part of standard medical care in China. If successful, our plan will expand to other transplanted organs and all regions of China.

## Trial status

Recruitment to the initial pilot study commenced Aug 2022. At the time of the initial journal submission, (Aug 2025), recruitment was ongoing using protocol version 1 (Jul 2022). No safety concerns existed to continue protocol-directed therapy and follow-up of all enrolled patients, with a current plan for database lock in Dec 2025.

## References

[1] Habre C Soccal PM Triponez F Aubert JD Krueger T Martin SP . Radiological findings of complications after lung transplantation. Insights Imaging. (2018) 9:709–19. doi: 10.1007/s13244-018-0647-9, PMID: 30112676 PMC6206387

[2] Waatevik M Johannessen A Gomez Real F Aanerud M Hardie JA Bakke PS . Oxygen desaturation in 6-min walk test is a risk factor for adverse outcomes in COPD. Eur Respir J. (2016) 48:82–91. doi: 10.1183/13993003.00975-2015, PMID: 27076586

[3] Walsh JR Chambers DC Davis RJ Morris NR Seale HE Yerkovich ST . Impaired exercise capacity after lung transplantation is related to delayed recovery of muscle strength. Clin Transpl. (2013) 27:E504–11. doi: 10.1111/ctr.12163, PMID: 23815281

[4] Hume E Ward L Wilkinson M Manifield J Clark S Vogiatzis I. Exercise training for lung transplant candidates and recipients: a systematic review. Eur Respir Rev. (2020) 29:200053. doi: 10.1183/16000617.0053-2020, PMID: 33115788 PMC9488968

[5] Candemir I Ergun P Kaymaz D Demir N Taşdemir F Sengul F . The efficacy of outpatient pulmonary rehabilitation after bilateral lung transplantation. J Cardiopulm Rehabil Prev. (2019) 39:E7–E12. doi: 10.1097/HCR.0000000000000391, PMID: 31241521

[6] Spruit MA Singh SJ Garvey C ZuWallack R Nici L Rochester C . An official American Thoracic Society/European Respiratory Society statement: key concepts and advances in pulmonary rehabilitation. Am J Respir Crit Care Med. (2013) 188:e13–64. doi: 10.1164/rccm.201309-1634ST, PMID: 24127811

[7] Rochester CL Alison JA Carlin B Jenkins AR Cox NS Bauldoff G . Pulmonary rehabilitation for adults with chronic respiratory disease: an official American Thoracic Society clinical practice guideline. Am J Respir Crit Care Med. (2023) 208:e7–e26. doi: 10.1164/rccm.202306-1066ST, PMID: 37581410 PMC10449064

[8] Wickerson L Helm D Gottesman C Rozenberg D Singer LG Keshavjee S . Telerehabilitation for lung transplant candidates and recipients during the COVID-19 pandemic: program evaluation. JMIR Mhealth Uhealth. (2021) 9:e28708. doi: 10.2196/28708, PMID: 34048354 PMC8213059

[9] Adam TJ Finkelstein SM Parente ST Hertz MI. Cost analysis of home monitoring in lung transplant recipients. Int J Technol Assess Health Care. (2007) 23:216–22. doi: 10.1017/S0266462307070080, PMID: 17493307

[10] Abidi Y Kovats Z Bohacs A Fekete M Naas S Madurka I . Lung transplant rehabilitation-a review. Life (Basel). (2023) 13:506. doi: 10.3390/life13020506, PMID: 36836863 PMC9962622

[11] Shearin S Braitsch M Querry R. The effect of a multi-modal boxing exercise program on cognitive locomotor tasks and gait in persons with Parkinson disease. NeuroRehabilitation. (2021) 49:619–27. doi: 10.3233/NRE-210218, PMID: 34806626

[12] Elnaggar RK Mahmoud WS Alsubaie SF Abd El-Nabie WA. Effectiveness of a multi-modal exercise program incorporating plyometric and balance training in children with hemiplegic cerebral palsy: a three-armed randomized clinical trial. Phys Occup Ther Pediatr. (2022) 42:113–29. doi: 10.1080/01942638.2021.1964674, PMID: 34396891

[13] Lu T Denehy L Cao Y Cong Q Wu E Granger CL . A 12-week multi-modal exercise program: feasibility of combined exercise and simplified 8-style tai chi following lung Cancer surgery. Integr Cancer Ther. (2020) 19:1534735420952887. doi: 10.1177/1534735420952887, PMID: 32851871 PMC7457649

[14] Gant KL Nagle KG Cowan RE Field-Fote EC Nash MS Kressler J . Body system effects of a multi-modal training program targeting chronic, motor complete thoracic spinal cord injury. J Neurotrauma. (2018) 35:411–23. doi: 10.1089/neu.2017.5105, PMID: 28795657 PMC6909697

[15] Daros Dos Santos T Pasqualoto AS Cardoso DM Da Cruz IBM Moresco RN Ferreira da Silveira A . Effects of multimodal exercise program on postural balance in patients with chronic obstructive pulmonary disease: study protocol for a randomized controlled trial. Trials. (2023) 24:532. doi: 10.1186/s13063-023-07558-9, PMID: 37580800 PMC10426202

[16] Torres-Sanchez I Valenza MC Saez-Roca G Cabrera-Martos I Lopez-Torres I Rodriguez-Torres J. Results of a multimodal program during hospitalization in obese COPD exacerbated patients. COPD. (2016) 13:19–25. doi: 10.3109/15412555.2015.1043428, PMID: 26418629

[17] Pison CM Cano NJ Cherion C Caron F Court-Fortune I Antonini MT . Multimodal nutritional rehabilitation improves clinical outcomes of malnourished patients with chronic respiratory failure: a randomised controlled trial. Thorax. (2011) 66:953–60. doi: 10.1136/thx.2010.154922, PMID: 21700760

[18] Cox-Flaherty K Moutchia J Krowka MJ al-Naamani N Fallon MB DuBrock H . Six-minute walk distance predicts outcomes in liver transplant candidates. Liver Transpl. (2023) 29:521–30. doi: 10.1097/LVT.0000000000000071, PMID: 36691988 PMC10101910

[19] Langer D Burtin C Schepers L Ivanova A Verleden G Decramer M . Exercise training after lung transplantation improves participation in daily activity: a randomized controlled trial. Am J Transplant. (2012) 12:1584–92. doi: 10.1111/j.1600-6143.2012.04000.x, PMID: 22390625

[20] Wu T Zhou S Wu B Chen J Zhu X Cai Y. The effect of early tracheal extubation combined with physical training on pulmonary rehabilitation of patients after lung transplantation: a randomized controlled trial. J Thorac Dis. (2022) 14:1120–9. doi: 10.21037/jtd-22-119, PMID: 35572910 PMC9096297

[21] Kerti M Bohacs A Madurka I Kovats Z Gieszer B Elek J . The effectiveness of pulmonary rehabilitation in connection with lung transplantation in Hungary. Ann Palliat Med. (2021) 10:3906–15. doi: 10.21037/apm-20-1783 33691452

[22] Pape L DeZwaan M Nohre M Klewitz F Tun EKT Prüfe J . A multimodal aftercare intervention improves the outcome after kidney transplantation - results of the KTx360 degrees aftercare program using claims data. EClinicalMedicine. (2024) 73:102652. doi: 10.1016/j.eclinm.2024.102652, PMID: 38841709 PMC11152610

[23] Qureshi R Gough A Loudon K. The SPIRIT checklist-lessons from the experience of SPIRIT protocol editors. Trials. (2022) 23:359. doi: 10.1186/s13063-022-06316-7, PMID: 35477436 PMC9044711

[24] Black R Nguyen DD Miles A Novakovic D Plit M MacDonald P . A pre- and post-operative protocol for assessment of voice and swallowing function in patients undergoing heart or lung transplantation. JHLT Open. (2025) 8:100261. doi: 10.1016/j.jhlto.2025.100261, PMID: 40491548 PMC12147448

[25] Choi EJ Kim W Jeon JY Ko EJ Yu J Choi SH . Intensive pulmonary rehabilitation in a pediatric lung transplantation patient: a case report. Medicine (Baltimore). (2021) 100:e25523. doi: 10.1097/MD.0000000000025523, PMID: 33907101 PMC8084064

[26] Hatt K Kinback NC Shah A Cruz E Altschuler EL. A review of lung transplantation and its implications for the acute inpatient rehabilitation team. PM R. (2017) 9:294–305. doi: 10.1016/j.pmrj.2016.09.013, PMID: 27721005

[27] Memme JM Erlich AT Phukan G Hood DA. Exercise and mitochondrial health. J Physiol. (2021) 599:803–17. doi: 10.1113/JP278853, PMID: 31674658

[28] Wang P Gao B Wang S Wang Z Zhao L Duan Y . Effectiveness of pulmonary rehabilitation on exercise capacity in adult patients with lung transplantation: a systematic review and single-arm meta-analysis. J Thorac Dis. (2024) 16:5727–41. doi: 10.21037/jtd-24-568, PMID: 39444878 PMC11494588

